# Bile duct injury after cholecystectomy: timing of surgical repair should be based on clinical presentation. The experience of a tertiary referral center with Hepp-Couinaud hepatico-jejunostomy

**DOI:** 10.1007/s13304-023-01611-7

**Published:** 2023-08-14

**Authors:** Felice Giuliante, Elena Panettieri, Agostino M. De Rose, Marino Murazio, Maria Vellone, Caterina Mele, Gennaro Clemente, Ivo Giovannini, Gennaro Nuzzo, Francesco Ardito

**Affiliations:** Hepatobiliary Surgery Unit, Fondazione “Policlinico Universitario A. Gemelli”, IRCCS, Università Cattolica del Sacro Cuore, Largo Agostino Gemelli 1, 00168 Rome, Italy

**Keywords:** Bile duct injury, Cholecystectomy, Hepatico-jejunostomy, Bile duct injury repair

## Abstract

Impact of timing of repair on outcomes of patients repaired with Hepp-Couinaud hepatico-jejunostomy (HC-HJ) after bile duct injury (BDI) during cholecystectomy remains debated. This is an observational retrospective study at a tertiary referral hepato-biliary center. HC-HJ was always performed in patients without sepsis or bile leak and with dilated bile ducts. Timing of repair was classified as: early (≤ 2 weeks), intermediate (> 2 weeks, ≤ 6 weeks), and delayed (> 6 weeks). 114 patients underwent HC-HJ between 1994 and 2022: 42.1% underwent previous attempts of repair at referring institutions (Group A) and 57.9% were referred without any attempt of repair before referral (Group B). Overall, a delayed HC-HJ was performed in 78% of patients; intermediate and early repair were performed in 17% and 6%, respectively. In Group B, 10.6% of patients underwent an early, 27.3% an intermediate, and 62.1% a delayed repair. Postoperative mortality was nil. Median follow-up was 106.7 months. Overall primary patency (PP) attainment rate was 94.7%, with a 5- and 10-year actuarial primary patency (APP) of 84.6% and 84%, respectively. Post-repair bile leak was associated with PP loss in the entire population (odds ratio [OR] 9.75, 95% confidence interval [CI] 1.64–57.87, *p* = 0.012); no correlation of PP loss with timing of repair was noted. Treatment of anastomotic stricture (occurred in 15.3% of patients) was performed with percutaneous treatment, achieving absence of biliary symptoms in 93% and 91% of cases at 5 and 10 years, respectively. BDI can be successfully repaired by HC-HJ regardless of timing when surgery is performed in stable patients with dilated bile ducts and without bile leak.

## Introduction

Bile duct injury (BDI) after cholecystectomy represents a potentially dramatic event, associated with significant morbidity and mortality. The rate of BDI after cholecystectomy ranges between 0.2 and 0.5% [1–5]. Notably, there was a peak in incidence after the introduction of laparoscopic cholecystectomy (LC) [6]. Nevertheless, the advent of LC has resulted in proven benefits as less postoperative pain, shorter hospital stay, improved cosmesis, and increased patient satisfaction. LC has been shown to be safe, but BDI occurs 3–5 times more often than with the traditional open approach [3–6]. Despite improvements in surgical training, increased experience with LC and the parallel developments in optics with high-definition cameras, the higher rate of BDI has remained stable over time. LC seems to be associated with more severe BDI, that is closer to the main biliary confluence and has a higher rate of associated vascular injury, compared to the open approach [7]. The best management of these patients requires a multidisciplinary team including surgeons, endoscopists, and interventional radiologists to allow the most efficient diagnostic and therapeutic strategy. When surgical repair is needed, a hepatico-jejunostomy (HJ) at the hilar plate according to the Hepp-Couinaud technique (HC-HJ) in a tertiary referral hepato-biliary (HB) center remains the best surgical repair of severe BDI [8, 9]. In selected cases, liver resection -with or without HJ- or liver transplantation may be required.

Many issues on the treatment of BDI after cholecystectomy remain unsolved. One of the most debated issues is the choice of the timing of surgical repair. Randomized controlled trials (RCTs) are not available on this topic and they are unlikely to be designed, given the extreme heterogeneity of several involved clinical aspects. Additionally, when reporting BDI repair results, many critical aspects need to be addressed: multiple definitions of timing of repair, complexity of concurrent factors, absence of standards to describe outcomes, and the frequent habit to combine results of previously repaired patients together with those of never repaired patients.

Aim of this study is to contribute to the discussion on the role of the timing of surgical repair of BDI after cholecystectomy by reporting short- and long-term outcomes of patients repaired with HC-HJ according to our institutional approach, with the purpose of finding predictive factors of failure of HC-HJ and to highlight the significant variables influencing the choice of timing of repair.

## Materials and methods

### Definitions

Severity of BDI was stratified according to the Strasberg classification [10]. The term “index repair” describes the definitive HC-HJ performed at our institution. All previous attempts at repair reported in the study, surgical or not surgical, were performed at referring institutions.

Timing of surgical repair after cholecystectomy was defined according to three categories: early repair (≤ 14 days), intermediate repair (> 14 days, ≤ 6 weeks), and delayed repair (> 6 weeks) [11].

The term “other bilio-enteric anastomoses” refers to every bilio-enteric anastomosis other than HC-HJ.

Post-operative complications were reported according to the Clavien-Dindo classification [12]. Grade ≥ 3 complications were considered as major morbidity. Long-term results, the definition of patency (primary and secondary), and its attainment and loss were evaluated according to the definition reported by Cho et al. [13].

### Patient characteristics

This is a retrospective observational study. All consecutive patients admitted for BDI after cholecystectomy at the HB Surgery Unit of the Fondazione Policlinico Universitario Agostino Gemelli, IRCCS, Catholic University, Rome, Italy, between January 1994 and March 2022 were analyzed. Data was obtained from a prospectively maintained database established at our institution.

Overall, 243 consecutive patients were admitted for BDI after cholecystectomy. A surgical treatment was performed in 129 patients (53.1%) as follows: 114 (88.4%) HC-HJs, 10 (7.8%) liver resections, 2 (1.5%) repairs over T-tube, 2 (1.5%) other bilio-enteric anastomoses, and 1 (0.8%) liver transplantation. The remaining 108 patients (44.4%) underwent non-surgical management of BDI and were treated by endoscopic retrograde cholangiopancreatography (ERCP) with sphincterotomy and stent placement, and/or percutaneous treatment (PTBD).

Six patients (2.5%) died before any attempt at repair. In this group, 1 patient had a type D injury, 1 patient had a type E3 injury, and 4 patients had a type E4 injury (associated with severe arterial injury in one case). When referred to our Unit, these patients were critically septic with multiple major complications (liver and renal failure, severe vascular injury, pulmonary complications, malnourishment) related to the late referral of the patients (range: 40–107 days from the cholecystectomy) and to the previous poor management of the BDI, including different failed attempts at repairing the injury.

**Fig. 1:**
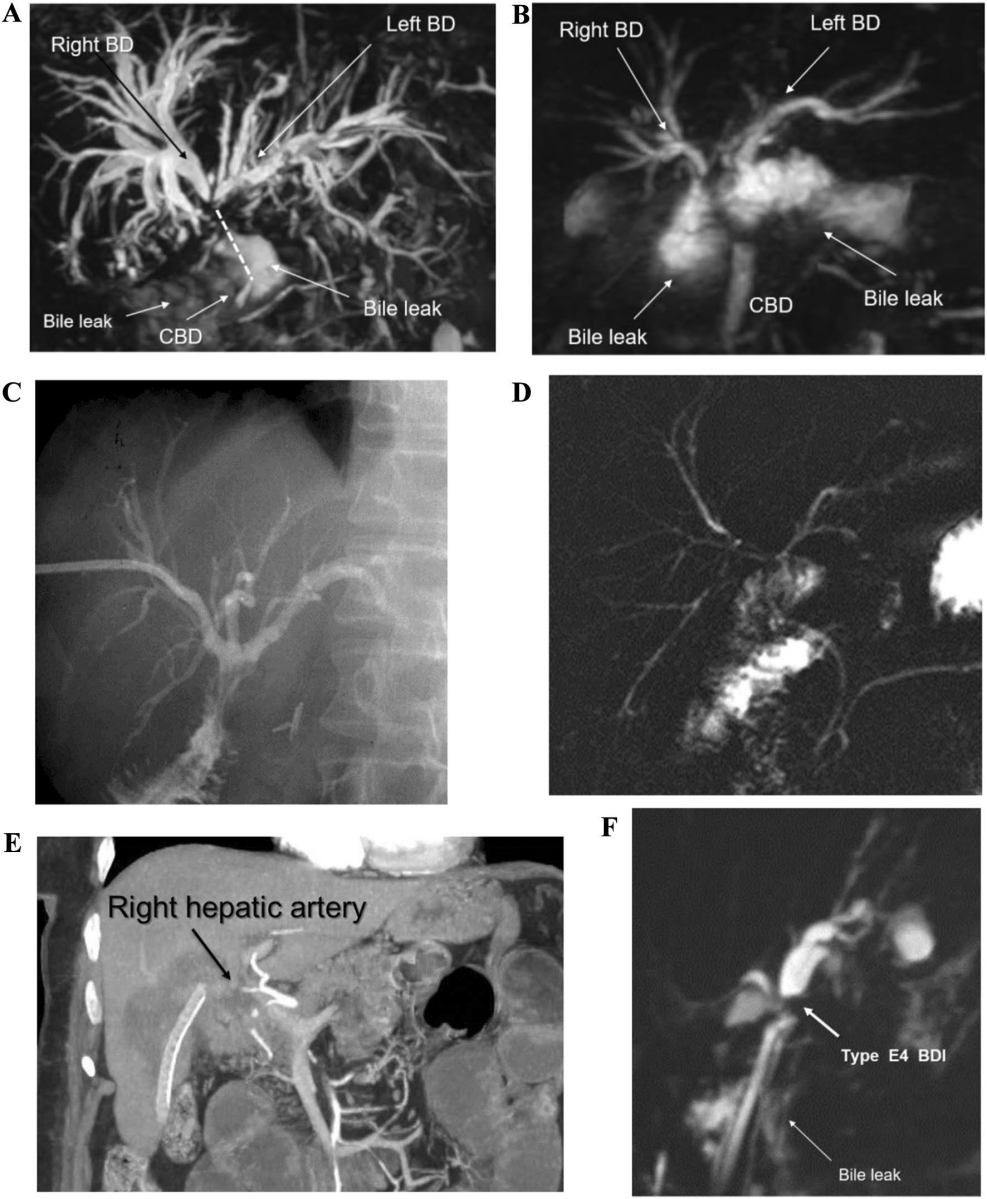
**A** MR cholangiography: Strasberg type E4 bile duct injury, with bile leak; the dotted line shows the long missing tract of the CBD. **B** MR cholangiography: Strasberg type E5 BDI, with bile leak. **C** Postoperative percutaneous cholangiography after HC-HJ. **D** MR cholangiography after HC-HJ. **E**, **F** MRI and MR cholangiography: right hepatic artery injury with Type E4 BDI with bile leak. *MR* magnetic resonance; *CBD* common bile duct; *BDI* bile duct injury; *HC-HJ* Hepp-Couinaud hepatico-jejunostomy; *MRI* magnetic resonance imaging

Among the surgical population, BDI was classified as type C in 2 patients (1.5%), as type D in 1 patient (0.8%), and as type E in 126 patients (93.8%): 24 E1-2, 76 E3, 18 E4, 8 E5 **(**Fig. 1a–b**)**. Among the 108 patients treated endoscopically and/or percutaneously, the following BDI types were noted: 26 type A, 8 type C, 26 type D, 48 type E (19 E1-2, 14 E3, 11 E4, 4 E5).

Demographic and clinical features of the 114 patients undergoing HC-HJ are shown in Table 1.

**Table 1:** Demographic and clinicopathologic characteristics of 114 patients who underwent Hepp-Couinaud hepatico-jejunostomy after bile duct injury

Characteristic	Entire cohort
	(n = 114)
Patient factors	
Age, median (IQR), yr	55 (43–66)
Sex, n (%)	
Male	45 (39.5)
Female	69 (60.5)
Clinical presentation at referral	
Biliary leak, n (%)	50 (43.8)
Jaundice, n (%)	39 (34.2)
Cholangitis, n (%)	25 (22.0)
Preoperative total bilirubin, median (IQR), mg/dL^a^	5.5 (1.5–13.4)
BDI factors	
Strasberg type, n (%)	
E1	1 (0.9)
E2	22 (19.3)
E3	75 (65.8)
E4	15 (13.1)
E5	1 (0.9)
Vascular injury, n (%)	8 (7)
Previous repair^b^	
Yes, n (%)	48 (42.1)
Hepatico-jejunostomy, n (%)	25 (52.1)
Repair over T-tube, n (%)	12 (25)
Suture without T-tube, n (%)	7 (14.6)
Endoscopic stenting treatment, n (%)	4 (8.3)
Timing of repair ^c^	
Early (< 2 weeks), n (%)	7 (6.1)
Intermediate (2–6 weeks), n (%)	18 (15.8)
Delayed (> 6 weeks), n (%)	89 (78.1)
Multiple jejunal anastomoses, n (%)	13 (11.4)
Short-term results ^c^	
90-day mortality, n (%)	0 (0)
90-day morbidity, n (%)	26 (22.8)
CD grade ≥ 3, n (%)	11 (9.6)
Bile leak, n (%)	8 (7)

*IQR* interquartile range; *BDI* bile duct injury; *CD* Clavien-Dindo

^a^Of the 114 patients, 113 patients were analyzed because data was unavailable for preoperative bilirubin in 1 patients

^b^It refers to a first attempt of repair performed at the referring institution

^c^It refers to the index repair performed at the referral center

### Surgical technique

According to the policy of our center, the repair was performed in patients in good general condition, with no signs of sepsis and no sign of bile leak. Therefore, an “early” repair was performed only in jaundiced patients with a complete stricture of the biliary tree, without bile leak at referral. On the other hand, an “intermediate” or “delayed” repair was performed after the resolution of the fistula in patients who presented with a bile leak. In patients with biliary fistula at presentation, the repair was delayed until the resolution of the bile leak. The closure of the biliary fistula (median time 50 days) was obtained with gradual abdominal drainage withdrawal, approximately 1 cm/7–10 days based on clinical condition and the output of the fistula, in an outpatient setting.

In all cases, a right subcostal laparotomy, extended to the xyphoid when necessary, was performed. One patient underwent a robotic-assisted HC-HJ. The dissection usually proceeds along the inferior surface of segment 4b up to the hilar plate in order to expose the main biliary confluence. The dissection plane lies away from the dense fibrous tissue involving the upper portion of the common bile duct (CBD) and the hepatic pedicle. Whenever the access to the hilar plate is too narrow to get a wide exposure, resection of the anterior portion of segment 4b is performed, obtaining a clear approach to the main biliary confluence and to the right secondary biliary confluence, if necessary. The left hepatic duct is longitudinally opened along its anterior wall toward the umbilical fissure to obtain a wide biliary stump. In case of a type E4 BDI, or if the stump of the CBD is too short, narrow, or the biliary tissue is extensively damaged by chronic inflammation, the right and left main bile ducts are further mobilized. Subsequently, the left hepatic duct is opened longitudinally toward the left, and the wall of the stump of the right hepatic duct is opened anteriorly and, when possible, sutured together with the left. This maneuver enables to create a single wider biliary stump and to perform the reconstruction through one single anastomosis in most cases. If needed, two or more hepatico-jejunal anastomoses can be created [14]. Usually, an interrupted suture with multiple absorbable 5/0 or 6/0 stitches is performed. The stitches are passed in full thickness through the biliary wall and in an extramucosal fashion through the jejunal wall, on a Roux-en-Y jejunal loop. Usually, a PTBD is placed the day before repair, preferably in the right biliary system, as an anatomical intraoperative landmark to help throughout the dissection. Subsequently, the PTBD is positioned through the anastomosis during the HC-HJ and left in place to drain it, allowing to perform postoperative cholangiography (Fig. 1c).

### Follow-up

Every patient underwent systematic follow-up including liver function tests (total and direct bilirubin, aspartate aminotransferase, alanine aminotransferase, alkaline phosphatase, and gamma-glutamyl transferase), transabdominal ultrasound (US) every 6 months, magnetic resonance imaging (MRI) once a year, and further radiologic examination if required (Fig. 1c–d). A telephone interview was done once a year to investigate the results of liver function tests and US, and to assess the clinical status of patients (i.e.: presence of symptoms, occurrence of cholangitis, jaundice).

### Statistical analysis

Continuous variables were compared using the T test, while categorical variables were compared using the χ2 test. Patency curves were generated using the Kaplan–Meier method. Univariable and multivariable analyses to identify factors associated with loss of patency were performed by using Cox proportional hazards regression models. Clinically relevant variables with *p* < 0.05 in univariable analysis were entered into each multivariable analysis. All statistical tests were two-sided, and *p* < 0.05 was considered statistically significant. Statistical analyses were performed with STATA software (version 14.0: StataCorp LLC, College Station, TX, USA).

## Results

Of 114 patients undergoing HC-HJ, 48 patients (42.1%) had already undergone previous attempts at repair -surgical or not surgical- at the referring institutions (Group A), while 66 (57.9%) had never been repaired before (Group B). In group A, 25 patients (52.1%) had undergone a previous HJ, 12 patients (25.0%) a surgical repair over T-tube, 7 patients (14.6%) a CBD suture without T-tube, and 4 patients (8.3%) an endoscopic treatment with biliary stents.

**Fig. 2:**
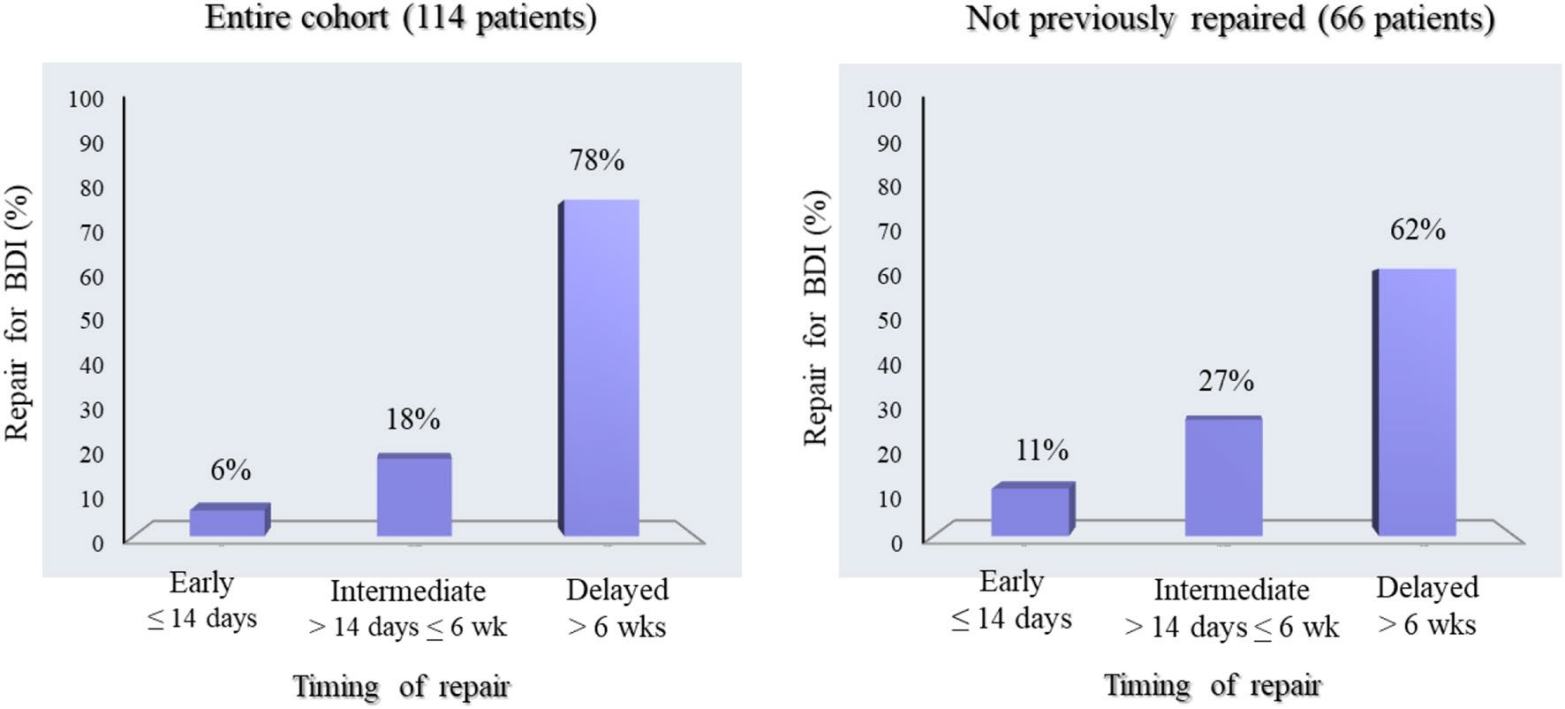
Histogram representing the distribution of timing of repair in the entire cohort and in not previously repaired patients. *BDI* bile duct injury; *wk* week

Regarding the timing of the index repair in the overall population, a delayed repair was most frequently performed. In particular, 89 patients (78.1%) underwent a delayed, 18 patients (15.8%) an intermediate, and 7 patients (6.1%) an early repair. Notably, among the 66 patients in Group B, 7 patients (10.6%) underwent an early index repair, 18 patients (27.3%) were repaired at an intermediate time interval, and in 41 patients (62.1%) a delayed repair was preferred (Fig. 2). Among these patients, a biliary fistula was present at referral in 36 cases (54.5%) and jaundice in 30 cases (45.5%).

In Group A all patients were repaired at a delayed interval and the overall median time of referral was 50 days (10–210).

The clinical characteristics of the entire cohort are summarized in Table 1.

### Short-term results

Ninety-day mortality in the overall surgical population was nil, with 26 patients (22.8%) developing 90-day post-operative morbidity, and a rate of major complications of 9.6% (11 patients). Eight patients (7.0%) had a post-operative bile leak.

### Long-term results

Long-terms results were available in 111 patients, with a median follow-up of 106.7 months (interquartile range 56.8–163.5).

**Fig. 3:**
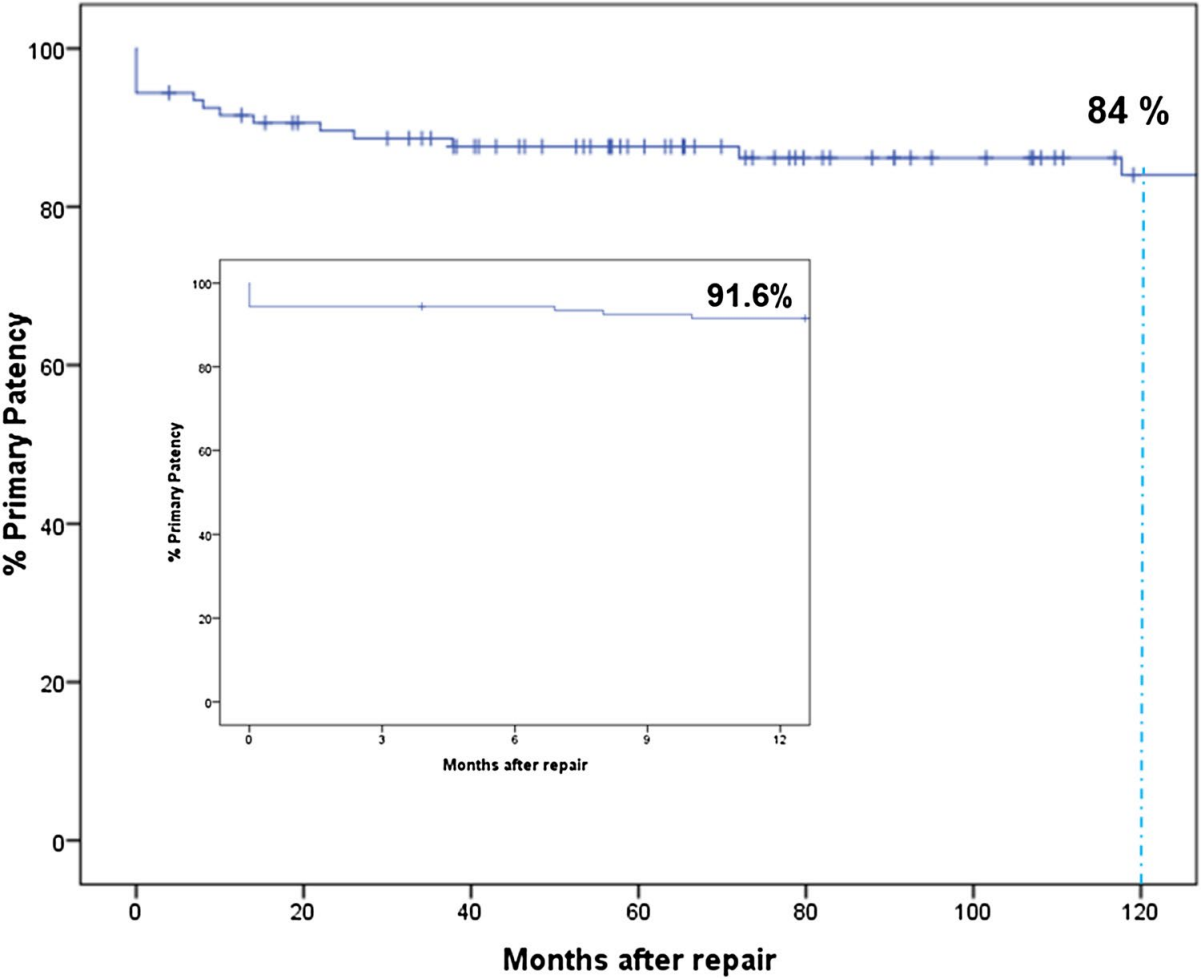
Actuarial primary patency rate in 111 patients undergoing index Hepp-Couinaud hepatico-jejunostomy. The box shows results within 1 year after repair

According to the definition of Cho et al. [13], the primary patency attainment rate after HC-HJ was 94.7%. The actuarial primary patency rate at 5 and 10 years was 84.6% and 84.0%, respectively (Fig. 3).

Overall, 21/111 patients (18.9%) developed cholangitis during the follow up, with evidence of an anastomotic stricture in 17 (15.3%), who were treated by a percutaneous approach. In particular, all patients underwent gradual percutaneous trans hepatic biliary stenting. This was based on the placement of a first PTBD of small size (usually 8 French), progressively replaced after 2–3 months with another drain of bigger size, up to reach a caliber of 20 French, which was maintained at least six months. Additionally, surgery was attempted in only one patient with the intent of creating a new HC-HJ. Nevertheless, surgery was eventually suspended, since the presence of extremely tight adhesions made it unsafe. The percutaneous treatment was successful at the first attempt in 7/17 patients (41.2%), while the remaining 10 patients underwent a failure of the first stenting treatment and required additional interventions (for some patients still ongoing at the time of the last follow-up).

In the overall population, absence of biliary symptoms was observed at 5 and 10 years in 87% and 81% of patients, respectively. Among patients needing percutaneous treatment of the anastomotic stricture, absence of biliary symptoms was obtained in 93% and 91% of patients at 5 and 10 years, respectively.

The following factors possibly involved in the loss of patency in patients submitted to HC-HJ were analyzed: sex, age ≥ 55 years, BDI injury type ≥ 3, associated vascular injury, previous attempt of repair, previous HJ, preoperative total bilirubin ≥ 5.5 mg/dl, post-repair bile leak, and timing of repair. After uni- and multivariable logistic regression analysis, only postoperative bile leak was associated with the loss of patency in the entire population (odds ratio [OR] 9.75, 95% confidence interval [CI] 1.64–57.87, p = 0.012). The loss of primary patency was not associated with the timing of repair (Table 2).

**Table 2:** Uni- and multivariable logistic regression analysis for loss of patency in 111 patients undergoing Hepp-Couinaud hepatico-jejunostomy for bile duct injury with available long-term follow-up

Factor	No. of patients	No. of events	Univariable analysis			Multivariable analysis		
			OR	95% CI	*p* value	OR	95% CI	*p* value
Patient factors								
Sex, male	43	11	1.99	00.76–5.20	0.158			
Age ≥ 55 years	56	15	2.99	1.06–8.40	0.038	2.09	0.70–6.26	0.186
Bile duct injury factors								
Strasberg type ≥ E3	84	18	2.18	0.59–8.07	0.243			
Associated vascular injury	8	1	0.59	0.07–5.10	0.634			
Repair factors								
Previous repair	48	10	1.24	0.48–3.22	0.653			
Previous hepatico-jejunostomy	25	4	0.77	0.23–2.55	0.673			
Preoperative bilirubin ≥ 5.5 mg/dL^a^	54	8	0.57	0.22–1.52	0.266			
Post-repair bile leak	7	5	13.75	2.45–77.11	0.003	9.75	1.64–57.87	0.012
Timing of repair								
Delayed	87	16	Ref	–	–			
Intermediate	17	4	1.36	0.39–4.74	0.624			
Early	7	1	0.74	0.08–6.58	0.787			

*OR* odds ratio; *CI* confidence interval; *Ref.* reference

^a^Of 111 patients, 110 patients were analyzed because data was unavailable for preoperative bilirubin in 1 patient

## Discussion

The best timing for BDI repair is still a matter of debate. The complexity of concurrent clinical factors and the low incidence of BDI make it difficult to design RCTs. The available data come from meta-analyses and large retrospective series, that however show extreme heterogeneity in terms of definitions of timing and outcomes, and of concomitant clinical factors.

A meta-analysis by Schreuder et al. [11] identified 2834 patients undergoing HJ for BDI from 21 studies, reporting 12 different definitions of timing of repair which make it difficult to compare results. However, the most commonly used timeframe was: immediate, early (< 2 weeks), intermediate (2–6 weeks), and delayed (> 6 weeks). Twelve studies, including 1875 patients, investigated the association between the BDI-HJ interval and postoperative morbidity, showing a higher risk of postoperative morbidity when the HJ is performed 3–6 weeks after the BDI, and the lowest risk after 6 weeks. Results from 15 studies including 1821 patients demonstrated a decreased risk of anastomotic stricture with the increase of the BDI-repair interval, with a lower risk of stricture after 9 weeks. No correlation between timing of repair and mortality was found (5 studies, 1046 patients). The conclusion of this meta-analysis was to avoid repair between 3 and 6 weeks after injury and a preference for delayed repair was reported.

According to a review and meta-analysis of 32 studies by Wang et al. [15], repair failure is more frequent after early repair than after delayed repair (31.9% vs. 17.1%, *p* < 0.001). In particular, the difference is more evident between repairs performed < 6 weeks and > 6 weeks (37.7% vs. 8.9%, *p* < 0.001). Postoperative bile leak (10.5% vs. 4.8%, p < 0.001) and need for revision surgery (23.9% vs. 4.0%, p < 0.001) were more frequent after early repair than after delayed repair. Amongst patients undergoing early repair, who had the least favorable outcomes, bile leak at presentation and surgical procedures different than HJ are more frequent than in the delayed group. Furthermore, repair performed by a HB surgeon is less common in this group of early repaired patients. All these results seem to suggest that the presence of a biliary fistula at the time of repair is a detrimental factor for successful outcomes and that a HJ -which is the optimal surgical repair procedure- not performed by an expert surgeon is another determinant factor for failure of early repair.

When a BDI is recognized intraoperatively, immediate repair is theoretically the best choice, with suture over T-tube, end to end anastomosis (with or without T-tube), and HJ being the options. However, the success of an immediate repair is related to the experience in biliary surgery of the surgeon. The same meta-analysis shows that immediate surgical repair performed by a HB surgeon has a significantly lower failure rate (18.9%) compared to that observed in the overall on-table repair (failure rate 60%) [15]. Moreover in the same study, it was clearly reported that early referral, compared with delayed referral, significantly decreased postoperative complications (*p* < 0.007) and biliary stricture (*p* < 0.001). To this regard, the Multi-society Practice Guidelines on prevention of BDI during cholecystectomy [16] state that, when a BDI is recognized (intra- or postoperatively), surgeons should refer the patient promptly to a hospital with a HB multidisciplinary team. If that is not achievable in a timely manner, it is highly recommended to consult an expert surgeon in BDI management.

A meta-analysis of 17 studies including 2155 patients, by Halle-Smith et al. [17] reported that it was not possible to perform a meta-analysis on post-HJ stricture according to the timing of HJ because of the heterogeneity of the proposed time intervals. Nevertheless, a trend towards a lower rate of HJ stricture by delaying the time of repair was noted. In the same paper, the authors showed that concomitant vascular injury (OR 4.96, 95% CI 1.92–12.86, *p* = 0.001), post-repair bile leak (OR 8.03, 95% CI 2.04–31.71, *p* = 0.003), and repair by a non-HB surgeon (OR 11.29; 95% CI 5.21–24.47; *p* < 0.001) were associated with a higher rate of anastomotic stricture, while there was no correlation with the Strasberg BDI grade (OR: 0.97, 95% CI 0.45–2.10, *p* = 0.93).

Different results seem to come from a European-African HepatoPancreatoBiliary Association (E-AHPBA) multi-center study [18] which analyzed 913 consecutive patients who underwent HJ after BDI. They identified three intervals to define the timing of reconstruction: early (day 0–7), intermediate (1–6 weeks), and late (6 weeks-6 months). Results demonstrated how the timing of HJ had no impact on anastomosis patency, 90-day re-intervention, and liver-related mortality. Nevertheless, biliary-related mortality and 90-day mortality were higher after early (3.5% and 2.4%, respectively) and intermediate (4.2% and 2.7%, respectively) than after late repair (1% and 0.6%; *p* = 0.041 and *p* = 0.137, respectively). In other words, mortality rates at early or intermediate timing were four times higher than that reported after delayed repair. Another limitation of this study is the relatively short mean follow-up, which was only two years. In fact, it is well known that the risk of developing anastomotic stricture remains a threat for several years after repair, and follow-up of these patients should be handled accordingly [9].

Regarding the risk of higher postoperative mortality rate after early repair, similar results were showed by Ismael et al., [19] who described a mortality rate associated with < 30-day repair significantly higher than after > 30-day repair (5% vs. 0%, *p* = 0.012).

Indeed, it could be argued that the timing of repair in itself should not be considered as an independent factor for the success of the repair. In fact, the choice of the timing of repair depends on several factors: complexity of BDI (i.e.: proximity to the main biliary confluence, associated vascular injury, diathermy injury), clinical presentation (i.e.: bile leak, cholangitis, jaundice), timing of BDI diagnosis, timing of patient referral, patient general condition (i.e.: sepsis, malnutrition), and surgeon experience in BDI repair. In our series the timing of repair had no impact on long-term results. This result is coherent with our policy of repairing the injury in a patient without biliary fistula, whenever occurs the closure of the fistula -median time of closure of the biliary fistula was 50 days- to avoid local inflammation as much as possible at surgery, and in absence of sepsis in all cases. The value of this experience is based on the fact that in our center this policy has remained the same over time. Moreover, the availability of a long median follow-up (106.7 months) seems to significantly increase the reliability of our results.

A delayed index repair was performed in 78.1% of patients in the entire population and was the most frequent approach also in non-previously repaired patients (62.1%). However, more than one third of not previously treated patients (38%) underwent an early or intermediate repair. This choice was made because these patients had already obtained closure of the bile leak at the time of referral, or they had never developed it in the first place (i.e.: jaundiced patients at presentation). With this policy, excellent immediate results were obtained. In fact, the overall 90-day mortality was nil. The absence of perioperative mortality is particularly important, since a mortality rate up to 5% is reported in the literature after surgical repair, and in particular in patients repaired at an early or intermediate time interval [19]. Indeed, in this series 6 patients (2.5%) died before any kind of treatment -surgical or non-surgical- mainly due to major septic complications associated with severe biliary and vascular injury, following late referral, and previous failed attempts at repair in non-HB centers.

Regarding long term results, primary patency was attained in 94.7% of patients. Additionally, the 5- and 10-year actuarial primary patency were high, 84.6% and 84.0%, respectively. Interestingly, neither a previous repair nor the timing of repair was associated with the loss of primary patency. After univariable analysis, age ≥ 55 years and the presence of post-repair bile leak were the factors associated with the loss of patency. After multivariable analysis, only postoperative bile leak remained an independent risk factor (OR 9.75, CI 1.64–57.87, *p* = 0.012). Nevertheless, given the highly limited number of events, the 95% CI was wide, and this result should be carefully evaluated. This result draws attention to the necessity of an extremely accurate surgical technique for repair, with the aim to reduce the risk for post-repair bile leak as much as possible. In this context, the use of the robot, in a stable patient and in a context of absence of local inflammation, could be of help for further improving long-term results of surgical biliary repair.

Another interesting result is the high rate of success of percutaneous treatment of anastomotic stricture using a progressively stenting dilation of the anastomosis, with a 93% and 91% of absence of biliary symptoms 5 and 10 years after the repair, respectively. This result highlights once again the necessity of a multidisciplinary evaluation of these patients in a center with high level expertise not only in HB surgery, but also in interventional radiology and advanced endoscopy.

The present analysis encompasses almost 30 years of experience of a tertiary referral HB center. Even though the surgical policy and approach have not substantially changed over time in our center, it is important to note that the great technical and technological developments in the field of interventional radiology and surgical endoscopy contributed to a better multidisciplinary management of patients with BDI. In particular, such improvements could successfully avoid surgical repair in several cases and facilitate the treatment of anastomotic stricture after surgery. As a result, the number of patients treated by non-surgical approach increased significantly over time, but for the patients in which surgery remained the treatment of choice, our approach remained the same.

Despite the limitations associated with this study, such as its retrospective nature and the relatively limited number of cases analyzed, some key points can be gained. In particular, when a BDI after cholecystectomy is not immediately repaired, according to the results of this study, we suggest to repair the injury in stable patients in good general condition (no signs of inflammation, infection, or sepsis), when the bile leak is resolved, preferably in patients with a dilated biliary tree, and when a vascular injury -if present- is well defined in its extension and actual severity. The appropriateness of this policy seems confirmed by the absence of postoperative mortality and by the excellent long-term outcomes. Nevertheless, further results from larger multicenter series and prospective multicenter registries -which should be implemented- are needed to further define the approach to these patients, who are in the majority of cases young and healthy people and therefore deserve extremely high attention and care.

## Data Availability

Data is not available.
